# Exploring the Equity Impact of Current Digital Health Design Practices: Protocol for a Scoping Review

**DOI:** 10.2196/34013

**Published:** 2022-05-17

**Authors:** Laura Evans, Jay Evans, Claudia Pagliari, Karin Källander

**Affiliations:** 1 Usher Institute University of Edinburgh Edinburgh United Kingdom; 2 UNICEF New York, NY United States; 3 Department of Global Public Health Karolinska Institutet Stockholm Sweden

**Keywords:** digital health, health equity, design, human-centered design

## Abstract

**Background:**

The field of digital health has grown rapidly in part due to digital health tools’ potential to reduce health inequities. However, such potential has not always been realized. The design approaches used in digital health are one of the known aspects that have an impact on health equity.

**Objective:**

The aim of our scoping review will be to understand how design approaches in digital health have an impact on health equity.

**Methods:**

A scoping review of studies that describe how design practices for digital health have an impact on health equity will be carried out. The scoping review will follow the methodologies laid out by Arksey and O’Malley, the Joanna Briggs Institute, and the PRISMA-ScR (Preferred Reporting Items for Systematic Reviews and Meta-Analyses extension for Scoping Reviews) checklist. The PubMed, Embase, Web of Science, and ACM Digital Library databases will be searched for peer-reviewed papers. The ProQuest Dissertations and Theses and Global Index Medicus databases will be searched for gray literature. The results will be screened against our inclusion and exclusion criteria. Subsequently, the data extracted from the included studies will be analyzed.

**Results:**

As of March 2022, a preliminary search of the peer-reviewed databases has yielded over 4900 studies, and more are anticipated when gray literature databases are searched. We expect that after duplicates are removed and screening is completed, a much smaller number of studies will meet all of our inclusion criteria.

**Conclusions:**

Although there has been much discussion about the importance of design for lowering barriers to digital health participation, the evidence base demonstrating its impacts on health equity is less obvious. We hope that our findings will contribute to a better understanding of the impact that design in digital health has on health equity and that these findings will translate into action that leads to stronger, more equitable health care systems.

## Introduction

### Background

Health is an essential human right. However, for every human to have access to this right, it is necessary to reduce the health inequities that plague low-resource and high-resource countries alike [1]. Improving digital health and the design of digital health tools has long been heralded as one of the many ways to tackle such inequities in part because of the increasing pervasiveness of digital technologies and in part because from its inception, digital health has been put forward to strengthen health care systems and improve accessibility [2-6]. However, health equity, digital health, and design are broad, even fuzzy, concepts that need clear definitions in order to truly begin to analyze and understand the possible impact of the design of digital health on health equity.

Health equity definitions have changed throughout the years, depending on the context to which these definitions were being applied [7-9]. In the 1980s, the World Health Organization commissioned a series of papers through their “Equity in Health” program, and the foundational definition of inequity was formed: “The term ‘inequity’...refers to differences in health which are not only unnecessary and avoidable but, in addition, are considered unfair and unjust” [10]. Throughout the 1990s and 2000s, different scholars and practitioners sought to refine this definition of *health equity* in order to identify measurable parameters and could therefore offer a higher degree of accountability and operationalization [7]. In 2018, Braveman et al’s [11] definition was able to consolidate the work of decades prior. For them, “health equity means that everyone has a fair and just opportunity to be as healthy as possible. Achieving this...means reducing and ultimately eliminating disparities in health and in the determinants of health that adversely affect excluded or marginalized groups” [11]. According to this definition, health equity is both a process and an outcome, providing 2 different points for measurement. The process is reducing and removing the obstacles that prevent marginalized and excluded groups from achieving health, while the outcome is the absence of such obstacles. For our scoping review, when seeking to assess the health equity impact that design methods for digital health tools have, health equity will be evaluated either as a process or as an outcome. As a process, the health equity impact can be assessed by analyzing if and to what extent the development and implementation of digital tools, through design processes, ameliorate or eliminate health inequities. As an outcome, the health equity impact can be assessed by analyzing if and to what extent design processes for developing and implementing digital tools establish or enhance opportunities for increasing health and eventually achieving full health.

Although some consider the use of the telephone in the 1980s as one of the first forms of digital health [12], it was not until the 2000s that the technologies and tools that are today understood as digital health (then called *eHealth*) emerged. Just as with the concept of health equity, digital health definitions have changed throughout the years, and even now, there are multiple widely used and cited definitions of *digital health* in peer-reviewed, gray, and white literature (Textbox 1).

**The most widely cited and currently used definitions of digital health.**

**World Health Organization definition [13]**

- “[T]he use of digital, mobile and wireless technologies to support the achievement of health objectives. Digital health describes the general use of information and communication technologies for health and is inclusive of both mHealth and eHealth.”

**Meskó et al [14] definition**

- “[T]he cultural transformation of how disruptive technologies that provide digital and objective data accessible to both caregivers and patients leads to an equal level doctor-patient relationship with shared decision-making and the democratization of care.”

**Kostova [15] definition**

- “Use of information and communications technologies to improve human health, healthcare services, and wellness for individuals and across populations.”

**Topol [16,17] definition**

- “The convergence of smartphone-enabled mobile computational and connectivity capabilities is only one aspect of digital medicine; it also encompasses genomics, information systems, wireless sensors, cloud computing, and machine learning that can all be incorporated into new systems of health management, built around real-world, patient-generated data.”

**Food and Drug Administration definition [18]**

- “The broad scope of digital health includes categories such as mobile health (mHealth), health information technology (IT), wearable devices, telehealth and telemedicine, and personalized medicine.”

**Healthcare Information and Management Systems Society definition [19]**

- “Digital health connects and empowers people and populations to manage health and wellness, augmented by accessible and supportive provider teams working within flexible, integrated, interoperable and digitally-enabled care environments that strategically leverage digital tools, technologies and services to transform care delivery.”

By reviewing the definitions above (Textbox 1), 3 main distinctives of digital health can be distilled. Digital health uses a vast array of digital technology; is used to improve health or prevent sickness; and is participatory in nature, meaning that patients and consumers are empowered to manage their health. For our scoping review, *digital health* will refer to any kind of tool that encompasses these three attributes.

A consensus exists—and is becoming stronger—that the role of digital health interventions is to increase existing access to health and strengthen health care systems [5,6,20]. It can be said that digital health interventions that widen the inequity gap, despite achieving positive results, fail as a whole because they neither increase the chances of individuals and communities to be healthy nor strengthen the health care system of which they are a part [20]. However, it would be simplistic to try to identify a single reason for why digital health interventions fail to reduce inequities. These interventions, which are complex in themselves, are deployed in complex environments (eg, multiple organizations, environments with competing priorities among stakeholders, etc) to address complex or “wicked” problems, such as reaching low-income populations to provide them with high-quality, affordable health care [3,21,22]. Not trying to understand the reasons behind why digital health interventions fail to reduce the inequity gap—or worse yet, why they widen it—would be unwise because this would perpetually hinder digital health from attaining its hallmark promise of reducing inequities across communities and among individuals through better and more accessible care [2-4]. For this reason, our research will focus on one of the characteristics of digital health technologies that is known to have a significant impact on equity—design [3].

Design, as a concept and as a field, has been gaining tremendous recognition in all spheres of life and work, touching “almost everything we experience today” and “[being] one of the most powerful forces in our lives, whether or not we are aware of it” [23]. Understanding how design affects processes, systems, objects, and people in the real world is the mission of countless businesses, nonprofits, and research organizations [24-26], yet in the incipient years of digital health, design was largely overlooked [27,28], resulting in significant resistance to use, the abandonment of technology, and detrimental health outcomes [29-31]. It has long been acknowledged that for digital tools and technologies to achieve their full potential, they need to be “people oriented,” and this is achieved by designing them to have human requirements, instead of technological ones, at their center [32]. Across the board, the use of design approaches that take users and other stakeholders into consideration in order to develop more person-oriented tools has been gaining significant momentum [33]. Early on, this kind of design was mostly known as *user-centered design*. However, throughout the years, the term *human-centered design* has gained more prominence and has come to replace the term *user-centered design* [34].

*Human-centered design* can be considered an umbrella term [3], which at times has resulted in the term being used as nothing more than a catchword [35]. In order to include a wide breadth of literature regarding the use of human-centered design for digital health but to guard against uses of human-centered design that are too vague, our scoping review will use the practical set of components that human-centered design interventions should exhibit, which are described by Holeman and Kane [3] as follows:

- Participatory co-design: There should be evidence that the people who will use new tools or be impacted by them have been included in a meaningful and clear way. They are an integral part of the team.
- Supporting or augmenting human skills: There should be evidence that new tools will serve the people in a way that empowers them in their job or environment. The purpose of technology is not just to increase efficiency or oversight over a group of people.
- Attending to human values throughout the course of an iterative design and implementation process: There should be evidence of genuine interest for a whole person and for that person’s circumstances. Purely technical issues do not drive implementation; instead, human values and technical requirements are considered in tandem and are refined and improved in a cyclical manner throughout the life of an implementation.

We believe that our research is relevant and necessary because it can contribute to the slowly growing body of evidence that shows how design practices in digital health have an impact on health equity by starting to bridge the gaps of knowledge between the assumed impact of digital health design on equity, the actual evidence of this impact in the literature, and the best design practices for helping to create more equitable health care systems.

### Aim and Research Questions

The aim of this scoping review will be to understand how design approaches in digital health have an impact on health equity.

Because of the wide-reaching nature of design, understanding the impact that design is currently having on equity and how design methods can help eliminate inequities is crucial. To this end, our research will seek to answer the following research questions:

- Research question 1: Is there evidence in the existing literature that design methods for digital health have an impact on health equity?
  How is the impact being measured?
  What are the long- and short-term impacts?
- Research question 2: In the existing literature, can common approaches be identified regarding how design methods can help reduce health inequities?
  If common approaches can be identified, can broad recommendations be made based on these approaches?

## Methods

### Scoping Review Method

Our research will follow the scoping review methodologies outlined by Arksey and O’Malley [36] and the Joanna Briggs Institute [37] to examine the current literature on how design practices are impacting equity in the field of digital health. The PRISMA-ScR (Preferred Reporting Items for Systematic Reviews and Meta-Analyses extension for Scoping Reviews) checklist [38] will also be used to ensure that methodological standards are followed. This methodology was chosen over a systematic literature review for 3 reasons. First, scoping reviews better allow for exploring all relevant concepts in the broad and diverse research area of equity in the context of digital health design [39]. Second, unlike systematic literature reviews, which seek to present a synthesis of evidence, scoping reviews collect, organize, summarize, and present results—an approach that is better suited for working with data derived from different kinds of studies (eg, qualitative studies, quantitative studies, and mixed methods studies) [36]. Third, scoping reviews allow for inclusion and exclusion criteria to be crafted and refined iteratively as the topic becomes better understood by the authors and more evidence is uncovered [37].

### Identifying Relevant Studies

The search strategy will be developed in a stepwise fashion, as seen in Textbox 2. This approach to developing a search strategy and searching the selected databases has been successfully used in other scoping reviews in the field of digital health [40-42].

**Steps for developing a search strategy.**

**Conducting searches in relevant databases**

- By using already known keywords and studies, preliminary searches of relevant databases will help expand and refine search terms.

**Searching key electronic, peer-reviewed databases and gray literature databases**

- PubMed, Embase, Web of Science, and ACM Digital Library will be used to search for peer-reviewed papers.
- For gray literature, ProQuest Dissertations and Theses and Global Index Medicus will be searched.

**Reviewing reference lists of relevant articles**

- Reviewing reference lists can help identify studies that may have been missed in previous searches. It can be useful for uncovering new search terms.

**Reaching out to experts through the Global Digital Health Network**

- Reaching out to experts can be useful for further identifying previously missed studies. The Global Digital Health Network is one of the most relevant and active networks of professionals of digital health.

Keywords related to *design*, *equity*, and *digital health* will be used for the searches. To further identify relevant keywords, preliminary searches of scientific databases and the internet have been conducted, and guidance from the librarian at the University of Edinburgh has been sought to further refine this search strategy. The full search strategy can be seen in Textbox 3.

**Search strategy.**

**Search terms**

1. *health equity/ or health services accessibility/ or socioeconomic factors/ or educational status/ or employment/ or unemployment/ or family characteristics/ or income/ or medical indigency/ or occupations/ or poverty/ or poverty areas/ or social change/ or social class/ or social conditions/ or digital divide/ or health disparity/*
2. Age Factors/ or exp Population Groups/ or sex factors/ or rural population/ or urban population/ or developing country/ or country economic status/ or (race or racial or ethnic* or urban or rural or age factor* or elderly or seniors or (old* adj2 (age* or adult* or people or person or patient* or men or women)) or gender).ti,ab,kf.
3. (age factor* or cities or (countr* adj4 (low or middle or income or develop*)) or demograph* or (determinant adj2 health) or (develop* adj2 (sustainab* or millennium or countr*)) or (digital adj1 divide) or disab* or disadvantag* or discrimination or dispari* or (dominant adj2 gender) or (impair* adj1 (visual* or hear*))).mp.
4. (economic* or (education* adj2 (status or attain* or level*)) or employment or equit* or ethnic* or gender or geograph* or housing or homeless* or illitera* or income or inequit* or last mile or (last adj1 mile) or literacy or literate or location or marginali* or migrant* or immigrant* or (minority adj2 (cultur* or religio* or ethnic* or racial)) or poor or poverty or race or racial or rural or (social adj1 (class or status)) or socioeconomic* or stigma or underserved or undocument* or unemploy* or vulnerable or wom#n).mp.
5. Terms 1 *or* 2 *or* 3 *or* 4
6. (co-creation or (co adj1 (creation or design)) or community based participatory research or community-based participatory research or (design adj2 (thinking or human or cent* or inclusive or participatory or service or user or experience or communit*))).mp.
7. *universal design/ or design for all/*
8. Terms 6 *or* 7
9. *telemedicine/ or telehealth/ or artificial intelligence/ or machine learning/ or medical informatics/ or electronic health records/ or mobile applications/ or exp Informatics/*
10. (artificial intelligence or digital health or e-health or ehealth or m-health or mhealth or (digital adj2 (health or solution* or system*)) or (health adj2 (electronic or record* or tele* or medical)) or ict4d or (information adj5 development) or machine learning or mobile health or telecare or telehealth or telemedicine or tele-health or teleconsultation or tele-consultation or tele-care or tele-medicine or (tele adj1 (medicine or care or health or consultation)) or ((virtual* or remote*) adj4 (visit* or consult* or meet* or appoint* or communicat*)) or (Health* adj4 tech*) or e-portal* or eportal* or (Patient* adj2 portal*) or (medical adj2 informatic*)).mp.
11. Terms 9 *or* 10
12. Terms 5
  *and* 8 *and* 11

### Selecting Studies

In order to select relevant studies, inclusion and exclusion criteria have been developed, as shown in Table 1. These criteria are structured according to the domains put forward by the Joanna Briggs Institute [37,39], which are “population,” “concept,” “context,” and “type of evidence.” The domain “other variables” has been added to capture language, date, and format criteria.

Although gray literature will be included in this scoping review, only 2 databases (ProQuest Dissertations and Theses and Global Index Medicus) will be searched. We acknowledge that this may prevent us from finding relevant data sources regarding human-centered design that may have been published as white papers or through more informal channels, such as blog posts or forums. However, we think that this is necessary to keep the number of articles under consideration manageable. On the other hand, the ACM Digital Library—the largest database for computing and information technology literature—has been included to widen the scope beyond health-related databases only. Additionally, it was decided that the cutoff date for inclusion will be 2009, as it is mostly after the early 2000s that digital health started to be more widely applied [43].

After removing duplicate studies, title screening will be done first. Studies that do not provide enough information in the title, along with those that appear to meet the inclusion criteria, will move forward to abstract screening. During abstract screening, the same approach will be taken, so that studies with abstracts that do not have enough information for determining if they meet the inclusion criteria will move forward to full-text screening along with those that clearly meet the inclusion criteria. Finally, full-text screening will be performed.

Two authors (LE and JE) will carry out screening. When consensus cannot be achieved regarding the exclusion or inclusion of specific studies, a third author (KK or CP) will screen the text to break the tie. Covidence software [44] will be used, as it provides a convenient workflow for screening and data extraction when working with multiple authors.

**Table 1:** Inclusion and exclusion criteria structured according to the “population,” “concept,” “context,” and “type of evidence” domains suggested by the Joanna Briggs Institute [37,39].

Domain	Inclusion criteria	Exclusion criteria
Population	Any population	N/A^a^
Concept	Focus on design in digital health that shows a direct (explicitly addresses health equity) or indirect (addresses related concepts such as the ones listed in the search strategy) impact on health equity Design methodology is used to develop or refine a digital health tool that is deployed at least to the pilot stage	Focus on digital health without evidence of a direct or indirect impact on health equity (eg, exclusively concerned with the impact on usability) Design methodology is not used to develop or refine a digital health tool that is deployed at least to the pilot stage (eg, design is used to establish the feasibility of creating a digital tool only)
Context	Any geographical or social context	N/A
Types of evidence sources	Peer-reviewed articles of any design Gray literature	Abstracts only Books Systematic literature reviews, scoping reviews, or protocols
Other variables	Published in English or Spanish Studies published on or after 2009 Full article is available digitally	Published in any language other than English or Spanish Studies published before 2009 Full article is not available digitally

^a^N/A: not applicable.

### Charting the Data

Once relevant articles have been selected, data will be extracted into the categories suggested by the Joanna Briggs Institute (Textbox 4). In an initial pilot step, 3 to 5 studies will be chosen, and relevant information will be extracted to understand if other categories for data extraction should be added.

**Joanna Briggs Institute key categories for data extraction [39].**

**Categories**

1. Author(s)
2. Year of publication
3. Origin/country of origin (where the source was published or conducted)
4. Aims/purpose
5. Population and sample size within the source of evidence
6. Methodology/methods
7. Intervention type, comparator, and details of these (eg, duration of the intervention)
8. Outcomes and details of these (eg, how measured)
9. Key findings that relate to the scoping review questions

### Ethics Approval

As required, our scoping review has obtained approval from the University of Edinburgh Ethics Committee. Because scoping reviews use secondary data, no further ethics approval is needed; however, we will review selected literature to ascertain whether they conducted their research according to ethical guidelines. The previously stated methodology establishes a transparent and reproducible search strategy and study selection inclusion criteria, which limit the potential for personal bias [45].

## Results

As of March 2022, a preliminary search of the peer-reviewed databases has yielded over 4900 studies, and more are anticipated when gray literature databases are searched. We expect that after duplicates are removed and screening is completed, a much smaller number of studies will meet all of our inclusion criteria. The results will be presented using a 2-fold approach. First, we will present a numerical overview of the amount and kinds of studies and the key themes of the studies. Second, we will write a narrative synthesis based on the evidence extracted.

## Discussion

### Study Implications

For our review, we will search for and analyze the research literature on health equity, digital health, and human-centered design that is available to date, with the aim of understanding how human-centered design approaches in digital health may have an impact on health equity. One of the anticipated findings is the misuse of the concept of human-centered design, as it is likely that in many instances, the concept is used just to define user requirements or evaluate usability instead of being applied as an overall guide for implementation (ie, from the planning stage to the final stages of scale-up and evaluation). At the core of our study, there is a desire to disseminate the findings as widely as possible among the digital health community, implementers, and researchers alike, in the hope that such findings can contribute to the better understanding of the role that design in digital health has in health equity. In turn, such understanding could translate into action that leads to stronger, more equitable health care systems.

### Conclusions

Although there has been much discussion about the importance of design for lowering barriers to digital health participation, the evidence base demonstrating its impacts on health equity is less obvious. As the digital health, design, and health equity fields continue to gain prominence in the sphere of health across all settings, we believe that scoping and analyzing the existing literature will be a useful exercise that will shed more light on the equity impact of digital health design practices.

## Abbreviations

PRISMA-ScR: Preferred Reporting Items for Systematic Reviews and Meta-Analyses extension for Scoping Reviews
